# Processing of Rhythm in Speech and Music in Adult Dyslexia

**DOI:** 10.3390/brainsci10050261

**Published:** 2020-04-30

**Authors:** Natalie Boll-Avetisyan, Anjali Bhatara, Barbara Höhle

**Affiliations:** 1 SFB1287, Research Focus Cognitive Sciences, Faculty of Human Sciences, University of Potsdam, Karl-Liebknecht-Str. 24-25, 14476 Potsdam, Germany; hoehle@uni-potsdam.de; 2 CNRS, (Integrative Neuroscience and Cognition Center, UMR 8002), Université de Paris, 45 rue des Saints-Pères, 75270 Paris, France; bhatara@gmail.com

**Keywords:** developmental dyslexia, Iambic/Trochaic Law, rhythmic grouping, musicality, speech perception, rhythm perception

## Abstract

Recent studies have suggested that musical rhythm perception ability can affect the phonological system. The most prevalent causal account for developmental dyslexia is the phonological deficit hypothesis. As rhythm is a subpart of phonology, we hypothesized that reading deficits in dyslexia are associated with rhythm processing in speech and in music. In a rhythmic grouping task, adults with diagnosed dyslexia and age-matched controls listened to speech streams with syllables alternating in intensity, duration, or neither, and indicated whether they perceived a strong-weak or weak-strong rhythm pattern. Additionally, their reading and musical rhythm abilities were measured. Results showed that adults with dyslexia had lower musical rhythm abilities than adults without dyslexia. Moreover, lower musical rhythm ability was associated with lower reading ability in dyslexia. However, speech grouping by adults with dyslexia was not impaired when musical rhythm perception ability was controlled: like adults without dyslexia, they showed consistent preferences. However, rhythmic grouping was predicted by musical rhythm perception ability, irrespective of dyslexia. The results suggest associations among musical rhythm perception ability, speech rhythm perception, and reading ability. This highlights the importance of considering individual variability to better understand dyslexia and raises the possibility that musical rhythm perception ability is a key to phonological and reading acquisition.

## 1. Introduction

Developmental dyslexia (henceforth, dyslexia) affects the acquisition of reading and writing skills despite adequate cognitive and motoric abilities and appropriate access to education. Beyond literacy, dyslexia is also characterized by deficits in spoken language processing, particularly in processing phonological information. For this reason, researchers have proposed that deficits in the processing of phonological information may be the bridge connecting the deficits in spoken and written language, e.g., [1,2,3,4]. One prominent theory of dyslexia proposes that phonological processing difficulties are a consequence of impaired auditory processing abilities, in particular when processing rhythm information in speech and music [5]. The present paper aims to connect these hypotheses by investigating the processing of one specific type of phonological information, namely, rhythm information in speech, and its potential associations with literacy and the ability to perceive musical rhythms in dyslexia.

### 1.1. The Phonological Deficit Hypothesis

The original phonological deficit hypothesis proposes that a deficit in phonological skills underlies dyslexia, as evidenced by difficulties with tasks that tap into phoneme awareness, letter-sound knowledge, verbal short-term memory, and rapid automatized naming. For a recent review see [6]. Research on this hypothesis has primarily concentrated on deficits regarding segmental (i.e., phoneme) information and has, for example, established that children with dyslexia do not seem to perceive phonemes in the same way as children without dyslexia. Specifically, they have a reduced sensitivity to phonemically relevant distinctions (e.g., when discriminating /p/ from /b/) and an enhanced sensitivity to allophonic variants (e.g., when discriminating different realizations of /b/) compared to listeners without dyslexia, who show clear effects of categorical perception of consonants (for a meta-analysis see [7]). As categorical perception is assumed to result from effects of the native language phonological system to speech perception, e.g., [8], weak categorical perception may indicate that the language-specific phoneme categories are not sufficiently well-established. In the case of dyslexia, less well-defined phoneme categories may create difficulties in the phoneme-grapheme mappings that are relevant for the acquisition and/or processing of written language.

### 1.2. Rhythm Perception Deficits in Dyslexia

More recent developments in dyslexia research have shown that the phonological deficits in dyslexia are not restricted to processing segmental information, but also affect the processing of suprasegmental (i.e., prosodic) information, and, in particular, the processing of rhythm. Rhythm is established by the regular occurrence of an element or a pattern in time. Rhythm is an important feature of languages’ prosody, and can be characterized by, for example, an alternation of more prominent (i.e., strong) syllables with less prominent (i.e., weak) ones. Languages differ in their rhythmic structure as the organization of speech in alternations of strong and weak syllables is determined by language-specific “metrical stress” rules [9,10,11]. For example, in English and German, the basic rhythmic unit has a strong-weak (i.e., trochaic) pattern, but in other languages such as Hebrew, the basic rhythmic unit is weak-strong (i.e., iambic) [9]. Compared to groups without dyslexia, groups of individuals with dyslexia show lower performance in tasks that require perceptual sensitivity to and/or knowledge of stress rules. For example, this is the case in discrimination tasks with words or phrases pronounced with correct or incorrect stress patterns, e.g., [12,13,14]–an effect that is even present in young children with a familial risk for dyslexia [15]. In addition, these abilities have been found to correlate with reading skills [16,17,18].

Goswami [5,19] has proposed that a fundamental deficit in the processing of rhythmic information is associated with dyslexia. This account focuses on the periodic modulations of amplitude (amplitude envelope) that are crucial to establish speech rhythm with amplitude peaks being aligned with the strong (stressed) syllables of a speech sequence and Goswami assumes that the processing of this amplitude envelope is impeded in dyslexia. These difficulties may result from atypical basic auditory processing: numerous studies have found that individuals with dyslexia show low performance in the perception of rise time (i.e., the velocity of the amplitude increase) and that the perception of rise time is related to the discrimination of word stress patterns (for a review see [20]. Research on the neural basis of this impairment suggests that in dyslexia, neural oscillations are not synchronized with auditory rhythms in the same way as in populations without dyslexia [21,22,23,24]. Independent of whether the basis of the impairment is perceptual or neural, according to Goswami, the problem in the processing of rhythm hinders the segmentation of speech into syllables and also the perception of subsyllabic units like rhymes and single phonemes, the latter case explaining the segmental phonological problems in dyslexia. Although at this point any causal interpretations of associations in neural rhythmic entrainment and dyslexia have to be taken with care, it is relevant to note that Goswami’s theory has the potential to account for a broader range of deficits that have been observed in dyslexia. Low performance in the perception of speech rhythm seems to extend to non-linguistic domains such as beat perception in music [25,26,27], and even to motor synchronization abilities such as rhythmic tapping [28,29,30], which suggests a domain-general rhythm processing deficit in dyslexia.

With its focus on rhythm processing, Goswami and colleagues’ work offers a substantial approach to the potential mechanisms underlying the performance of individuals with dyslexia in different domains. In our study, we intend to broaden the view on rhythm perception in dyslexia by looking at duration and intensity as acoustic cues of speech rhythm perception. Acoustically, strong and weak syllables can be distinguished on the basis of specific cues such as intensity, duration, and pitch, with strong syllables often being louder, longer, and higher than weak ones [31,32]. Interestingly, these different cues have different effects on rhythmic grouping and segmentation: while alternations in syllables’ duration lead to the perception of weak-strong patterns, alternations in intensity and pitch lead to the perception of strong-weak patterns [9,33,34,35,36,37,38]; for more details see 1.3. The main goal of this paper is to investigate rhythmic grouping according to this bias in individuals with dyslexia.

Of course, not only speech is rhythmically structured. Rhythm is a domain-general phenomenon. Similar organizational rhythmic principles with regular alternations of strong and weak elements are also found in music [9,39], where the same acoustic cues (intensity, duration, and pitch) are relevant for conveying rhythm, and the same tendency to use these cues differently at the beginning or the end of a unit is often exhibited [39,40,41]. If rhythm perception in speech and music relies on shared perceptual mechanisms or shared rhythm representations, then it should be the case that individuals with better music abilities should also show enhanced language abilities [42,43,44]. In line with this, [45] reported that, within a group of adults with dyslexia, musicians outperformed non-musicians on several auditory measures, including rise time, frequency, intensity, and timing perception, even reaching the same levels of performance as musicians without dyslexia. However, the advantage that musicians with dyslexia experienced in the auditory perception tasks did not extend to their literacy and phonological awareness. Accordingly, other researchers doubt that dyslexia relates to poor rhythm perception, e.g., [46]. A second goal of the present paper, therefore, is to further examine whether rhythm processing deficits in speech and music are linked with reading deficits in dyslexia.

### 1.3. Biases on Auditory Rhythmic Grouping

In this paper, we will investigate for the first time how biases on auditory rhythm perception affect speech rhythm perception by adults with dyslexia. For this, we focus on rhythmic grouping of speech following the Iambic/Trochaic Law (ITL) [9]. According to the ITL, rhythmic perception is guided by universal biases. These biases have the effect that sequences of sounds varying in intensity tend to be perceived as trochees (strong-weak), whereas sound sequences varying in duration tend to be perceived as iambs (weak-strong; e.g., [33,34,35,36]). These biases have been attested for speakers of various languages, including English [33,34,36,47,48], German, French [38,49], Spanish [48], and Italian [37]. Since rhythmic grouping preferences are asymmetrical between the perceived acoustic cues, these biases cannot simply be accounted for by a tracking of acoustic cues to prominence in the signal. Importantly, asymmetries in rhythmic grouping are mirrored in the rhythm structures in language and music where final prominence is usually marked by a long syllable or note, and initial prominence by a loud syllable or beat, which supports the assumption of the ITL as universal [19]. More recent research, however, indicates that rhythmic grouping preferences are subject to individual variation and depend to some degree on aspects such as individuals’ language background [38,47,48,49,50] and their musical abilities [51,52]. 

#### 1.3.1. Effects of Language Background on Rhythmic Grouping

Language background’s effects on perception may relate to differences in the function of stress between the languages: [38] hypothesized that when perceiving speech, the ability to draw on abstract phonological representations of lexical stress would facilitate German speakers’ rhythm processing. This is because German uses lexical stress contrastively (e.g., /’te,nor/ ‘common sense’ vs. /te’nor/ ‘singer’), while French does not. In [38]’s rhythmic grouping experiment, German and French listeners listened to syllable streams, in which syllables alternated in intensity (loud-soft-loud-soft…), pitch (high-low-high-low…), duration (long-short-long-short…), or neither (flat control condition). Participants were asked to indicate via button presses whether they perceived strong-weak or weak-strong groupings. The result was that both groups perceived iambs and trochees as predicted by the ITL, but the German listeners were more consistent and had clearer rhythmic grouping preferences than the French listeners. Moreover, German but not French listeners experienced the illusion of hearing strong-weak groupings when listening to the control sequences that did not contain any acoustic cues to rhythm. Ref. [38] argue that this effect is likely to also be driven by the presence of abstract phonological representations of stress in German: As words in German are pre-dominantly trochaic, German listeners might apply a default grouping to sound sequences based on their linguistic experience. Since French has no lexical stress, there may be no reason for a default grouping based on their experience. 

#### 1.3.2. Effects of Musical Background on Rhythmic Grouping

Musical ***experience*** as defined by the number of acquired musical instruments, the duration of musical training, and the earliest age of acquiring a musical instrument has been found to influence rhythmic grouping. However, this seems to be modulated by the individuals’ language background and has, to this point, only been found to affect native speakers of French and not native speakers of German [38,49,51,52]. French speakers who are musically experienced have clearer preferences for grouping acoustically complex non-speech sounds [49] as well as for grouping speech, though only if they are also proficient speakers of German [51]. While general musical experience never predicted monolingual German speakers’ grouping of speech, their ability to perceive musical rhythm as measured by a standardized musical ability test (the Musical Ear Test, henceforward MET [53]) did (though their ability to perceive melodies did not) [52]. Musical abilities can be, but not necessarily, correlated with musical experience [46]. Instead, they may relate to more general auditory perception abilities, which vary widely among individuals [29]. In addition, the abilities to perceive and discriminate musical rhythms do not always correlate with musical melody perception abilities [54]. Together, these results suggest a specific connection between language and music via rhythmical properties. 

### 1.4. Hypotheses and Predictions

Individuals with dyslexia have repeatedly exhibited relatively weak stress and rhythm processing abilities, even in domains other than language (e.g., in tapping and music perception). This suggests that their rhythmic grouping preferences will also be weak, especially since rhythmic grouping depends on native language phonological knowledge. Given the findings that musical ability also influences rhythmic speech grouping, the present study set out to investigate the relations among speech rhythm processing, musical rhythm perception ability, reading ability, and dyslexia in German listeners. We aimed at investigating the following research questions:

(1) Do adults with dyslexia have less consistent rhythmic speech grouping preferences than adults without dyslexia?
(2) Do adults with dyslexia show lower musical rhythm perception abilities than adults without dyslexia?
(3) Does musical rhythm perception ability predict rhythmic speech grouping in dyslexia?
(4) Does musical rhythm perception ability predict reading ability in dyslexia?

We hypothesized the following:

(1) Based on the hypothesis that adults with dyslexia have difficulties in processing rhythm, we expect them to show weak grouping preferences. Hence, they should show less asymmetrical grouping preferences when hearing sequences varying in intensity or duration than adults without dyslexia. Further, if this rhythmic deficit hinders the establishment of phonological representations for metrical structure, adults with dyslexia should not show grouping preferences when hearing rhythmically invariant sequences.
(2) We assumed that musical rhythm perception ability would be lower in individuals with dyslexia than in individuals without.
(3) If rhythm perception in music and speech share cognitive underpinnings, we expect that higher musical rhythm perception ability would be associated with more consistent preferences in rhythmic grouping of speech in adults with dyslexia.
(4) If reading difficulties are linked with general underlying difficulties with rhythm processing, then musical rhythm perception ability should predict reading ability in dyslexia.

To investigate these hypotheses, we conducted a rhythmic grouping experiment with adults with and without dyslexia and measured their musical rhythm ability by means of the MET [53], and their reading ability by means of the Salzburger Lese- und Rechtschreibtest SLRT-II [55]. In order to avoid pre-selecting or grouping participants based on their musicality and cognitive abilities, we applied regression modeling for data analysis, with musical rhythm ability, musical experience, and cognitive abilities as covariates.

## 2. Materials and Methods

### 2.1. Participants

Participants were 23 monolingually-raised adult native speakers of German with dyslexia (nine women, 14 men, mean age = 24 years, age range: 17–35 years) and 23 (12 women, 11 men) age-matched controls. An additional participant with dyslexia was raised bilingually, and, hence, excluded together with the age-matched control. Participants gave informed consent before taking part.

The inclusion criterion for participants with dyslexia was that they showed us their formal testimonial of their developmental dyslexia diagnosis. In Germany, there are no nation-wide standards for dyslexia diagnosis. To verify the diagnosis provided by the participants, we compared how the groups with and without dyslexia fared at a reading test (i.e., the SLRT-II; [55], see below). Results of a linear regression indicated significantly lower nonword reading ability for the group with dyslexia compared to the group without (β = 41, SE = 6.06, t = 6.77, *p* < 0.001). This result allowed us to conclude that the testimonial of the dyslexia diagnosis did justify the division of the participants into two groups (with vs. without dyslexia). Hence, we used group (rather than reading ability scores) as a factor to test assumptions regarding dyslexia. Other than these, there were no further constraints on recruitment. Participants of both groups were recruited in the cities of Berlin and Potsdam by means of distribution of flyers and online advertisements on social media, to make sure that our sample would not only consist of university students. For a detailed summary of the groups’ background information and the groups’ average performance in the tasks described in Section 2.2, see Table 1.

The sample size is justified, as effect sizes of prior rhythmic grouping studies were high: for example in [38] for comparisons between French and German listeners in the intensity condition Cohen’s d = 1.4 (large) and Cohen’s d = 1.1 (large) in the duration condition; for comparisons between conditions (duration vs. intensity, and duration vs. control) within native speakers of German Cohen’s d = 4.4 (large). Moreover, we tested our design using the PANGEA software (https://jakewestfall.shinyapps.io/pangea/, see [56]), which revealed a high power (0.91) for a study design including a four-way interaction with 23 participants per group with the alpha level set at 0.05, and an assumed medium effect size of 0.45. This effect size of 0.45 is conservative given the large effect sizes found in prior studies, however, since prior studies on rhythmic grouping have, as yet, not included adults with dyslexia, power calculations have to be taken with caution.

### 2.2. Task Battery

#### 2.2.1. Rhythmic Grouping Preferences

In order to assess rhythmic speech grouping preferences, we used the stimuli and procedure from [37], Experiment 1. The stimuli were 90 speech-like streams that consisted of different simple syllables in which one consonant was always followed by one vowel (e.g., /…zulebolilozimube…/). The streams were text-to-speech synthesized with a German pronunciation and flat F0. There were three conditions: An intensity condition in which every second syllable was louder than the preceding one, a duration condition in which every second syllable was longer than the preceding one, and a control condition, in which all syllables were of equal intensity and duration. The task was to listen to each of the nonsense speech streams and to indicate by button press whether this pattern consisted of strong-weak or weak-strong disyllables. The proportion of strong-weak responses in the three conditions (intensity/duration/control) served as a dependent variable (Section 3.1/Section 3.3); for details, see [38].

#### 2.2.2. Musical Rhythm Perception Ability

Receptive musical rhythm abilities were assessed using the Musical Ear Test [53]. Participants heard 52 pairs of rhythmic sequences, which are containing 4–11 wood block beats, and had to decide whether the two sequences were the same or different. The obtained proportion of correct responses was used as a dependent measure to evaluate whether the group with dyslexia showed lower performance than the group without dyslexia (Section 3.2). Furthermore, this measure was used as an independent variable to understand its role as a predictor of rhythmic grouping (Section 3.3) and reading ability (Section 3.4).

#### 2.2.3. Questionnaire

An interview based on a questionnaire was used to collect information on the participants’ musical and language background, and, if applicable, their dyslexia status and therapy experience (for details and a summary of the results, see Table 1). Questions were read out by the experimenter, who also filled out the questionnaire based on the responses. Following [49,51,52], a predictor of musical experience was extracted using the answers to questions regarding the number of acquired instruments, the age of acquiring the first instrument, and the duration of years of musical practice. In the following analyses, it was tested whether this predictor was correlated with musical rhythm ability (Section 3.2), rhythmic grouping (Section 3.3), and reading ability (Section 3.4).

#### 2.2.4. Reading Ability

Participants completed the reading fluency test of the Salzburger Lese- und Rechtschreibtests (SLRT-II) [55], a standardized test for the diagnosis of dyslexia. They were asked to read aloud lists of words and nonwords within a time limit (one minute per list). It allows for a separate diagnosis of deficits in automatic word recognition versus synthetic sound-based reading. The latter is predicted to be particularly weak in individuals with dyslexia. Note that we did not use this test to diagnose any of the participants with dyslexia. The purpose of this test was to verify that the groups defined on presence vs. absence of formal diagnosis of dyslexia truly differed in reading ability (see Section 2.1), and to test whether musical rhythm ability predicted reading ability (Section 3.4).

#### 2.2.5. Cognitive Ability

Many studies suggest that individual variability in cognitive abilities such as general verbal comprehension, short-term memory, and processing speed can influence performance in psycholinguistic experiments (for a systematic review, see [57]). In order to verify that potential differences between the groups with or without dyslexia in the experimental task are not due to differences in such general cognitive abilities, participants completed four subsets from the Wechsler Adult Intelligence Scale WAIS-IV (a version adapted for German, [58]), a standardized tool for determining the intelligence quotient. To test verbal comprehension (specifically, verbal reasoning and semantic knowledge), participants performed the subtest Similarities, in which participants heard 18 pairs of words (e.g., piano & drum, or friend & enemy), for which they had to describe which attributes they share. Next, to test short-term memory, they performed subtests that measured their digit span. Specifically, they listened to sequences of orally presented numbers, and, in three subsequent sub-tests, they were required to repeat them in as heard, backward, or in sequential (ascending) order. 

For measuring their processing speed, we selected two subtests: Symbol search and Coding. In Symbol Search, participants were required to search for two target symbols in a row of different symbols, and to indicate whether the target symbols were present or not. In Coding, nine different numbers (1–9) are assigned a different symbol. In the task, participants are presented with a list of numbers and are required to draw the corresponding symbol next to each of the numbers. (Participants additionally completed a nonword repetition task for adults [59], which was based on the Mottier test, a standardized test for German-speaking children [60]. Because of redundancy with the digit span tests (Section 2.2.5), which also test verbal memory, we did not include the data of the nonword repetition test in the analyses.) A composite score of the results of all subtests served as a covariate in analyses of rhythmic grouping (Section 3.1/Section 3.3), musical rhythm perception ability (Section 3.2) and reading ability (Section 3.4).

### 2.3. Data Processing and Analyses

For the analyses, we included data from both the groups of adults with dyslexia (N = 23) and without dyslexia (N = 23). The analysis (Section 3) consisted of four parts. 

First, to address hypothesis (1), we tested whether rhythmic speech grouping preferences by the two groups (with vs. without dyslexia) differed from chance in the three acoustic conditions (intensity, control, and duration) by means of generalized linear mixed-effects (Section 3.1). 

Second, to address hypothesis (2), a linear regression analysis with the MET scores as a dependent variable was performed in order to determine whether group differences existed, while controlling for general cognitive ability, and musical experience (Section 3.2).

Third, to address hypothesis (1) and (3), we tested whether rhythmic grouping preferences differed between groups, and whether it depended on individuals’ musical rhythm perception ability. For this, we performed a generalized linear mixed-effects model analysis. In a stepwise fashion, we incrementally increased the models’ complexity to understand the effects of the factors group (which we predicted to have an effect) and musical rhythm perception ability (which we predicted to have an effect) on the three conditions (intensity, duration, control), while, ultimately, controlling for general cognitive ability and musical experience. Our method was to compare mixed-effects models that either included or excluded predictors to find the combination of predictors that accounted for most variance in the data, following the recommended procedures [61,62,63] (Section 3.3). 

Fourth, to address hypothesis (4), we assessed the association of musical rhythm perception ability and reading ability in both the group with and the group without dyslexia, while again controlling for musical experience and cognitive abilities. For this, we performed a linear regression analysis with nonword reading ability (i.e., SLRT nonword reading scores) as the dependent variable, and group, musical rhythm perception ability, cognitive ability and musical experience in the fixed part (Section 3.4). 

For the control variable “cognitive ability,” a composite score was generated that combined the averaged WAIS-IV subtest scores. For the control variable “musical experience”, we generated a composite score on the basis of three questions from the questionnaire representing the participants’ years of musical training, their age of beginning musical training, and the number of learned musical instruments/activities. Both composite scores were created by means of Principal Component analysis (see Appendix A, Table A1) to avoid collinearity. Collinearity occurs when a number of independent variables are correlated, which poses a problem to regression analyses. Principal component regression is a commonly used method to reduce collinearity, as it eliminates the dimensions that are causing the collinearity problem [64] (p. 446). Following the classical procedures, we included the first principal components (PCs) as independent factors in our subsequent regression analyses. The first PC reflecting cognitive ability accounted for 58% of the variance contained in the data of the 4 WAIS-IV subtests, which were represented by this PC to a comparable degree (see Appendix A for details). The first PC reflecting musical experience accounted for 82% of the variance of the 3 questions that were equally represented by this variable (see Appendix B, Table A2).

All analyses were performed in R [65] using the package lme4 [66]; graphs were generated using the package ggplot2 ([67]). For plotting modeled data, the package effects [68] was used to extract the model estimates and respective *SE*s.

## 3. Results

### 3.1. Rhythmic Grouping Preferences

**Figure 1:**
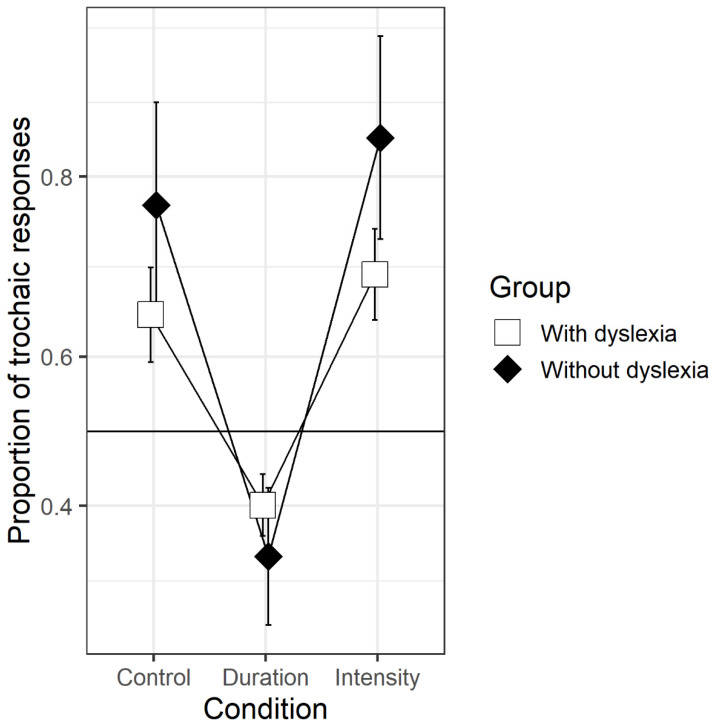
Proportions of trochaic responses (back-transformed, y-axis adjusted to the logit space) in the three acoustic conditions for both groups. The graph reflects the estimates of a simple logit linear mixed-effects model (responses ~ condition * group + (condition + 1||participants) + (1|items)…).

Tests against chance (see Appendix C, Table A3) revealed that in both the intensity and control condition, trochaic (strong-weak) responses were above chance for both the group with dyslexia (intensity: *p* < 0.001; control: *p* = 0.03) and the group without dyslexia (both *p*’s < 0.001). In the duration condition, both groups gave more iambic (weak-strong) responses than expected by chance (both *p*’s < 0.001, see Figure 1). 

### 3.2. Musical Rhythm Ability

We compared how the two groups (with vs. without dyslexia) fared at the MET for rhythm, while controlling for general cognitive ability and musical experience. Control participants’ average rhythm MET scores were higher (73.49% correct, SD = 9.96) than those by participants with dyslexia (61.87% correct, SD = 9.92) with large effect size (Cohen’s d = 1.17). Results of a linear regression confirmed that differences between groups were significant (*p* = 0.03). Moreover, rhythm MET scores were significantly predicted by cognitive ability (*p* < 0.01), but not by musical experience (*p* = 0.28, for the full results, see Appendix D, Table A4). Groups did not, however, differ with regards to their musical experience (β = 0.77, SD = 0.46, t = 1.68, *p* = 0.1). This suggests that dyslexia is associated with reduced musical rhythm perception ability that is independent of musical experience.

### 3.3. Predictors of Rhythmic Grouping Preferences

Next, we tested whether rhythmic grouping preferences differed in strength between groups and explored the role of individual differences in musical rhythm perception ability, cognitive ability, and musical experience. For this, we report the main results of all models that entered our stepwise regression analysis. To measure the consistency of rhythmic grouping preferences (i.e., how consistent participants were in grouping duration variation as iambs and intensity as trochees), we entered contrasts between conditions into our models. In all models (see Appendix E, Table A5, Table A6, Table A7, Table A8 and Table A9), significant effects were obtained in the Duration-Intensity contrast and in the Control-Duration contrast (both *p*’s < 0.001), indicating that in both the intensity and control condition, more trochaic responses were given than in the duration condition. 

**Figure 2:**
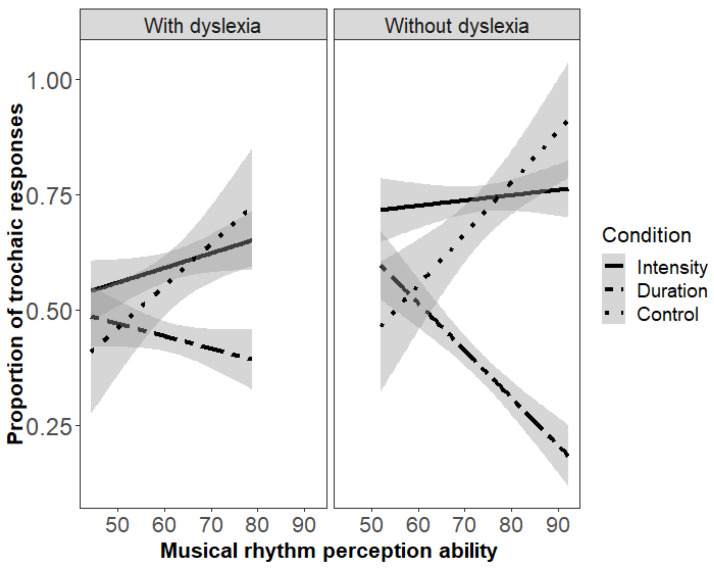
Linear regression lines illustrating the effects of musical rhythm perception ability (Musical Ear Test scores) on rhythmic grouping in the three acoustic conditions separated by group (left panel: with dyslexia, right panel: without dyslexia).

Model 1, serving as a basis, included only the interaction of Condition and Group in the fixed part (Formula: Response ~ Condition/(Group) + (1 + Duration-Intensity + Control-Duration || participant) + (1 | item) to test the hypothesis that adults with versus without dyslexia differ in their rhythmic speech grouping preferences. Model results (fully reported in Table A5 and depicted in Figure 2) show significant group differences in both the intensity (*p* = 0.003) and the control condition (*p* = 0.02), with more trochaic responses by adults without dyslexia than by adults with dyslexia. In the duration condition, no group differences were found. 

Model 2 included only the interaction of Condition and Musical rhythm perception ability in the fixed part, excluding group (Formula: Response ~ Condition/(Musical rhythm perception ability) + (1 + Duration-Intensity + Control-Duration || participant) + (1 | item) to evaluate the general contribution of musical rhythm perception ability on rhythmic speech grouping preferences. Model results (see Table A6) show significant effects of musical rhythm perception ability on all conditions: higher musical rhythm perception ability was associated with more trochaic groupings in the intensity (Intensity*Musical rhythm perception ability: *p* = 0.04) and control condition (Control*Musical rhythm perception ability: *p* < 0.001), and more iambic groupings in the duration condition (Duration*Musical rhythm perception ability, *p* < 0.001, see Figure 2). Model comparisons revealed that Model 2 was a better fit than Model 1 (χ^2^ = 39.53, *p* < 0.001).

Model 3 included the three-way interaction of Condition, Group and Musical rhythm perception ability in the fixed part (Formula: Response ~ Condition/(Group* Musical rhythm perception ability) + (1 + Duration-Intensity + Control-Duration || participant) + (1 | item) to understand whether group and musical rhythm perception ability predict speech grouping preferences independently. Results (see Table A7 and Figure 2) suggest that this is not the case. Interactions of Group with the Intensity and Control conditions that were present in Model 1 no longer reached significance in Model 3, and the interaction of Duration*Group did not reach significance either. However, the interactions Duration*Musical rhythm perception ability (*p* < 0.001) and Control*Musical rhythm perception ability (*p* < 0.001) that were present in Model 2 remained highly significant in Model 3. This suggests that group differences in the Control condition as attested in Model 1 are due to differences in musical rhythm perception ability between the groups. Moreover, the results suggest that variance in the Duration and Control condition is better captured by differences among individuals’ musical rhythm perception ability than by dyslexia status. There were no three-way interactions of any of the conditions with group and musical rhythm perception ability. Model comparisons revealed that Model 3 was a better fit than Model 2 (χ^2^ = 13.62, *p* < 0.001).

Two further models tested the potential effects of two control variables: cognitive ability (Model 4, reported in Table A8) and musical experience (Model 5, reported in Table A9). These models revealed the same effects that were also present in Model 3. However, because neither of these control variables significantly influenced participants’ grouping preferences in any of the conditions, nor did an inclusion of these factors improve the model fit, we do not discuss these models further (detail and model outputs are provided in Table A8 and Table A9). 

To summarize, Model 3 (Table A7), which included interactions of condition with group and musical rhythm perception ability, accounted best for the data, which revealed effects of musical rhythm perception ability on the control and duration (but not the intensity) condition, but no significant effects of the group factor. 

### 3.4. Predictors of Nonword Reading Ability

**Figure 3:**
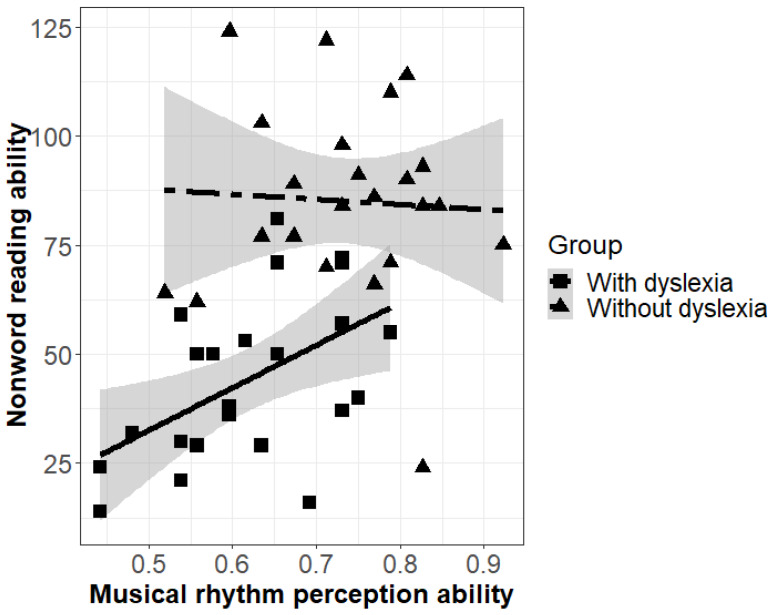
Linear regression lines reflecting the association between nonword reading ability (Salzburger Lese- und Rechtschreibtests (SLRT) scores) and musical rhythm perception ability (Musical Ear Test (MET) scores) split by group, shades indicate confidence intervals, rectangles (with dyslexia) and triangles (without dyslexia) the individuals’ averages.

Results of a linear regression analysis (see Appendix F, Table A10 for details) revealed neither effects of cognitive ability nor of musical experience on reading ability (no main effect, no interaction). There was, however, a significant main effect of group, indicating that—as expected—the group without dyslexia had higher reading ability than the group with dyslexia (*p* < 0.001). Moreover, there was a marginal interaction of musical rhythm perception ability and group (*p* = 0.056, Cohen’s f^2^ = 0.10 (medium)). To understand the interaction, we tested the effect of musical rhythm perception ability on reading ability per group. Results were that musical rhythm perception ability positively predicted reading ability by individuals with dyslexia (β = 97.77, SE = 35.76, t = 2.73, *p* = 0.01, Cohen’s f^2^ = 0.36 (large)) but not by individuals without dyslexia (β = −11.693, SE = 48.25, t = −0.24, *p* = 0.81; see Figure 3).

## 4. Discussion

The present study is based on the theory that there is a deficit in rhythm processing in dyslexia [1,2,3,4], which we studied by exploring the modulating effects of musical rhythm perception ability on rhythmic speech grouping by adults with and without dyslexia. Populations with dyslexia have previously been demonstrated to have difficulties with processing stress and rhythm information [12,13,14], which suggests that the phonological deficit affects not only segmental but also suprasegmental aspects of speech. Hence, we investigated whether adults with dyslexia have reduced abilities in rhythmic grouping of speech, an ability that has previously been found to depend on native language phonological knowledge [38,47,48]. Rhythm, however, is not only part of phonology, but is also an integral aspect of other auditory domains, such as music. Previous studies have established that there are links between individuals’ musical abilities and their rhythm processing [52]. Hence, we hypothesized to find links between rhythm processing in speech and music and reading ability in dyslexia.

Specifically, our research was intended to provide answers to the following questions, and delivered the following central findings:

(1) Do adults with dyslexia have less consistent rhythmic speech grouping preferences than adults without dyslexia? Our results indicate that this is not the case, as adults with dyslexia show clear rhythmic speech grouping preferences (Section 3.1), and rhythmic grouping preferences are not different between adults with versus without dyslexia (Section 3.3).
(2) Do adults with dyslexia have lower musical rhythm perception abilities than adults without dyslexia? Our results (Section 3.2) suggest that this is the case.
(3) Does musical rhythm perception ability predict rhythmic speech grouping in dyslexia? Our results suggest that this is the case: Musical rhythm ability predicted rhythmic grouping preferences (Section 3.3).
(4) Does musical rhythm perception ability predict reading ability in dyslexia? Our results suggest that this is the case: We found that musical rhythm perception ability predicts reading ability in dyslexia (Section 3.4).

The results are discussed below. 

### 4.1. Dyslexia, Rhythmic Grouping Preferences, and Musical Rhythm Ability

First, regarding the link between dyslexia and rhythmic speech grouping, results revealed significant preferences for groupings as predicted by the ITL in all conditions (iambic in the duration condition, trochaic in both the control and intensity condition), by native speakers of German with and without dyslexia. This result was unexpected. Our original hypothesis was that rhythmic grouping preferences would be weakened in dyslexia. This hypothesis was motivated by results from prior studies showing that individuals with dyslexia have weakened stress perception abilities, e.g., [12,13,14]. In prior studies, we found that native speakers of French had weakened rhythmic grouping preferences compared to native speakers of German—a result argued to relate to differences in the phonological systems of German and French (due to the lack of contrastive lexical stress in the French language). Since French speakers have, moreover, repeatedly been found to have weakened stress perception abilities, e.g., [69,70], and the same is true for individuals with dyslexia, e.g., [12,13,14], we drew a parallel. Unexpectedly, results attested that German speakers with dyslexia show consistent grouping preferences at the group level, just like German speakers without dyslexia. This result replicates our previous findings with native speakers of German without dyslexia and extends them to native speakers of German with dyslexia.

It is important to further explore why listeners with dyslexia generally show the same pattern of responses as those without in the rhythmic speech grouping task: At first glance, this conflicts with the assumption that individuals with dyslexia predominantly have a deficit in rhythm processing. However, the present results can be better understood by considering the results of the model comparisons that addressed the fourth research question about the association of dyslexia, rhythmic speech perception, and musical rhythm perception ability. Model 1 (Table A5, the baseline model that excluded the musical rhythm perception ability factor), suggested a detrimental impact of dyslexia on rhythmic speech perception in both the intensity and control condition. This is in line with prior studies that suggested links between speech rhythm perception and dyslexia, e.g., [12,13,14]. 

Importantly, these effects disappeared when, in Model 3 (Table A7, as well as Models 4 and 5 (Table A8 and Table A9) that additionally controlled for cognitive ability and musical experience), musical rhythm perception ability was added as a predictor. This suggests that differences in individuals’ musical rhythm perception ability better capture the variance in the data than the individuals’ dyslexia status (i.e., there are individuals with dyslexia who have high musical rhythm perception ability with consistent grouping preferences, and individuals without dyslexia with low musical rhythm perception ability with inconsistent grouping preferences). However, even though this suggests that rhythmic speech perception is independent of dyslexia and only modulated by musical rhythm perception, adults with dyslexia had overall lower musical rhythm perception ability than adults without dyslexia, which implies an indirect effect of group on rhythmic speech perception. 

Notably, as predicted, musical rhythm perception ability accounted for variance in the rhythmic grouping of duration-varied and rhythmically invariant control speech sequences, but, in contrast with our predictions, not of intensity-varied speech sequences. These findings suggest that the relation between musical rhythm perception ability and speech rhythm processing cannot be simply explained by a general deficit in perceiving the acoustic information that is relevant for perceiving rhythm (otherwise the processing of intensity-varied speech sequences should also be related to musical rhythm perception ability). The results of the control condition, in which acoustic cues to rhythm were absent, suggest that the relation between musical rhythm perception ability and speech rhythm processing is established via phonological knowledge. In previous studies [38], it was proposed that German listeners might perceive trochees even in the absence of acoustic cues to rhythm, because German has trochaic metrical stress and their abstract knowledge about this phonological property of their language affects German listener’s perception (as commonly seen also in sound perception which is also affected by native language phonemic categories). Ref. [52] also observed that musical rhythm perception ability was associated with this default grouping procedure, and it was speculated whether individual differences in basic auditory perception abilities might lead to differences in how listeners establish phonological knowledge. The fact that the present study replicates the effect of the connection between musical rhythm perception ability and default trochaic groupings with adults with dyslexia is interesting, as it offers additional support for the interpretation that that default rhythm perception procedures are subject to individual variation and are associated with more general auditory rhythm perception abilities. 

In order to explain why musical rhythm perception ability, contrary to our predictions, did not affect grouping of intensity-varied sequences by adults with dyslexia, we consider previous studies on the ITL. Infants have been found to use pitch and intensity cues for trochaic groupings (pitch: [37,71], intensity: [72]) more readily than duration cues for iambic groupings [37,71,72]. Based on these findings it has been proposed that the use of duration cues for grouping is acquired, while the use of other rhythmic cues for trochaic groupings is innate (more evidence for this proposal comes from studies with rats [73,74] but c.f. [75,76] for evidence that the use of duration for iambic groupings is also innate). The present finding that intensity-based grouping is unmodulated by musical rhythm perception ability is consistent with the assumption of an innate preference for trochaic groupings when intensity alternations are perceived. Speculatively, this might suggest that in dyslexia, perception of innately biased speech processing routines is unimpaired. It would be interesting if future studies followed up on this. 

### 4.2. Dyslexia and Musical Rhythm Ability

Regarding the associations among dyslexia, reading ability and musical rhythm perception ability, results were as predicted: First, adults with versus without dyslexia differed in their musical rhythm perception ability: as a group, adults with dyslexia showed a lower performance in the MET rhythm subtest than the control group. This result is in line with previous findings indicating deficits in musical rhythm processing abilities in dyslexia [25,26,27]. However, it must be noted that performance in the MET is associated with short-term memory performance [58]. This is in line with literature that has found short-term memory ability to be enhanced in musical people [77,78,79]. However, it is also well-known that groups with dyslexia have less efficient short-term memory abilities than groups without dyslexia (see Table 1, it is also true for the present sample: WAIS, Working Memory: digit span: with dyslexia: 24 (15–38); without dyslexia: 28.86 (22–37)), and it is debated whether this reduced short-term memory efficiency is the basis of the impairment or is an effect of the phonological deficit [3,80]. In fact, we found that musical rhythm ability was predicted by cognitive ability, a composite variable that included the participants’ digit span scores. In order to better understand if musical rhythm ability is lower in dyslexia independently of related cognitive abilities, future studies should aim to control for this confounding factor. 

### 4.3. Dyslexia, Nonword Reading Ability, and Musical Rhythm Ability

We tested whether reading ability was predicted by group and by musical rhythm perception ability. As expected, the groups differed in their reading ability. Interestingly, we furthermore found a marginally significant interaction (*p* = 0.056) of group and musical rhythm perception ability to account for reading ability. We explored this interaction (although results based on this insignificant interaction have to be taken with care), and found that, in particular, reading ability of adults with dyslexia was predicted by musical rhythm perception ability: the lower their performance in the MET rhythm subtest, the lower was their score in the nonword reading test. This finding is consistent with prior studies, which found links between musical rhythm perception ability and reading ability (e.g., [81] and references therein). Both these results support theories of links between general rhythm processing abilities and dyslexia and, accordingly, with reading ability.

Note that the lack of a relation between musical rhythm perception ability and reading ability in adults without dyslexia does not justify the conclusion that this association is exclusive to dyslexia. Potentially, a link between musical rhythm perception ability and reading ability could also be found if a reading test were used with adults without dyslexia that elicits greater variability in this groups’ reading ability than the SLRT, a test particularly designed for identifying dyslexia in adulthood. Moreover, the relation between musical rhythm perception ability and reading ability may be non-linear, with ceiling effects of musical rhythm perception ability at a certain level of high reading ability that adults without dyslexia typically reach. It will be interesting to address these questions in future research. 

## 5. Conclusions

In sum, the main findings of the present study are the following: First, rhythmic grouping of speech is not predicted by dyslexia status, but by musical rhythm ability. That is, the present study does not provide direct evidence for the theory that there is a specific speech rhythm processing deficit in dyslexia. However, the fact that we found the group of adults with dyslexia to show lower musical rhythm perception ability than the group of adults without dyslexia, and that musical rhythm ability predicted speech rhythm grouping indicates that there is a link between rhythm processing in music and speech and dyslexia. Second, musical rhythmic skills predict reading in dyslexia. The results suggest clear links between dyslexia (i.e., reading ability), musical rhythm perception ability, and speech rhythm processing, not only when rhythmic cues are available but also when a lack of cues triggers knowledge-driven default processing routines. All in all, the results point to individual differences in the group of adults with dyslexia that are explained by their musical rhythm perception ability. 

The present findings cannot inform about causal relationships between musical rhythm perception ability and dyslexia. However, they raise the possibility that rhythm perception ability is a key to phonological and reading acquisition. The present results are in line with two assumptions about the underlying reasons for these links. The first assumption is that deficits connected with dyslexia can be compensated by rhythm perception ability. The second assumption is that the deficits connected with dyslexia are a consequence of lower rhythm perception ability. That is, potentially, individuals with lower rhythm perception ability have a higher risk for developing phonological and reading deficits. 

Future studies should address the question of how musical rhythm perception ability, speech rhythm perception, and reading are causally connected. To explore the first assumption, studies should assess the potential of rhythmic interventions in dyslexia therapy, and therewith, follow a line of research that has already been initiated, e.g., [82,83]. Ideally, future research should explore pre-/post-test paradigms to explore whether musical rhythm perception ability can be enhanced by training. This can then be extended to other types of rhythmic behavior, such as motor synchronization (e.g., tapping) with rhythmic beats, to pave the way for targeted rhythm-based therapeutic approaches. To explore the second assumption, future research should conduct longitudinal studies with very young infants with a familial risk for dyslexia (for a similar suggestion, see [84]), to pave the way for our understanding of whether the ability to perceive rhythm in music (and other sensory domains) is a reliable early marker of developmental dyslexia.

## Figures and Tables

**Table 1:** Summary of the results of all questions from the questionnaire as well as all musical and cognitive tests for both the group of adults with versus without dyslexia.

General Participant Information (in N)	With Dyslexia (N = 23)	Without Dyslexia (N = 23)
Age (mean, range)	23.781 (7–35)	23.95 (18–35)
Gender	9 women, 14 men	12 women, 11 men
Handedness	19 right, 1 left, 3 both	22 right, 1 left
Native language = German	23	23
Mother with native language other than German	1	2
Father with native language other than German	3	0
Vision problems (short- or far-sighted, usually compensated by glasses)	10	9
Hearing problems	Auditory perception disorder (1), Otitis media with effusion in childhood (1), Un-defined (1)	0
Language problems	Stuttering (1), Specific Language Impairment (1)	0
Learning problems	Attention Deficit Hyperactivity Disorder (2)	0
**Highest Education (in N)**		
Without degree	1	1
Hauptschule	0	0
Realschule	7	2
Fachhochschulreife	2	0
Hochschulreife (Abitur)	8	13
Berufsausbildung	8	1
Hochschulabschluss	0	7
Promotion	0	0
Other	1	0
**Foreign Language Experience (Max. Number of Learned Foreign Languages, in N)**		
One	8	-
Two	9	11
Three	5	7
Four	1	3
Five	0	2
**General Musical Experience (in N)**	19 yes, 4 no	18 yes, 5 no
**Specific Musical Experience (Average, Range in Bracket)**		
Number of instruments (or musical activities such as choir, dance)	2.05 (1–6)	2.94 (1–6)
Age of first musical instrument or activity acquisition	10.72 (4–24)	8.35 (4–20)
Years of practicing a musical instrument or activity	6.11 (1–16)	13.06 (1–30)
Hours spent singing per week	3.74 (0–35)	2.18 (0–10)
Hours spent dancing per week	0.72 (0–6)	1.09 (0–7)
Hours spent with instrument play per week (excl. participants without musical experience)	4.26 (0–40)	2.53 (0–20)
Hours spent listening to music per week	13.04 (0–80)	13.86 (0–40)
**Musical Abilities (Self-estimated, Likert Scale 0 (No Ability)–10 (Perfect) (Average, Range in Bracket)**		
Musical instrument (excl. participants without musical experience)	3.6 (0–9)	5.35 (0–9)
Dancing	1.9 (0–8)	3.82 (0–9)
Singing	2.59 (0–7)	4.55 (0–9)
**Preferred Music Styles (in N)**		
Classical music	8	11
Pop	13	15
Rock	16	14
Hiphop	14	7
Jazz	7	7
Popular folk (Schlager)	2	1
Reggae	11	3
Techno	8	7
Heavy Metal	4	4
World music	5	3
Country	2	4
Other	1 (dubstep)	1 (child music)
**Dyslexia Therapy (in N)**		
Received dyslexia therapy	20 of 23	
Therapy included music therapy	3 of 23	
**Dyslexia Characteristics (in N)**		
Reading and writing difficulties	9 of 23	
Reading difficulties alone	3 of 23	
Writing difficulties alone	11 of 23	
**Performance in Musicality Tests (in % Correct Responses, Range)**		
Musical Ear Test: rhythm test	62% (44–79)	74% (52–92)
**Performance in Cognitive Tasks (in N Correct Responses, Range)**		
Salzburger Lese-Rechtschreib-Test: word reading	85.13 (18–119)	127.82 (92–156)
Salzburger Lese-Rechtschreib-Test: pseudoword reading	44.13 (14–81)	85.82 (24–124)
Wechsler Adult Intelligence Scale, Verbal Comprehension: similarities	23.13 (14–33)	26.77 (14–34)
Wechsler Adult Intelligence Scale, Working Memory: digit span	24 (15–38)	28.86 (22–37)
Wechsler Adult Intelligence Scale, Processing Speed: symbol search	37.04 (20–56)	43.18 (25–65)
Wechsler Adult Intelligence Scale, Processing Speed: coding	64.83 (34–96)	81.14 (63–115)

## Appendix A

Principal Component Analysis was used to generate one composite score to reflect general cognitive ability on the basis of four tests from the WAIS. In the analyses, we used the first Principal Component as a factor to represent cognitive ability, which, as can be seen from the loadings in Table A1, represented all four variables to a comparable degree, and captured a proportion of 0.58 of their variance.

**Table A1:** Results of the Principal Component Analysis over the data of four variables relating to general cognitive ability.

Principal Component Analysis	Comp.1	Comp.2	Comp.3	Comp.4
**Importance of Components**				
Standard deviation	1.53	0.87	0.85	0.43
Proportion of Variance	0.58	0.19	0.18	0.05
**Loadings**				
Similarities (verbal comprehension)	0.45	0.57	0.60	0.33
Digit span (short-term memory)	0.40	−0.76	0.49	−0.13
Symbol search (processing speed)	0.58	0.26	−0.29	−0.72
Coding (processing speed)	0.54	−0.19	−0.57	0.60

## Appendix B

Principal Component Analysis was used to generate one composite score to reflect musical experience on the basis of three questions from the questionnaire regarding the number of acquired musical instruments/activities, the earliest age of acquiring a musical instrument/activity, and the duration of musical training in years. In the analyses, we used the first Principal Component as a factor to represent musical experience, which, as can be seen from the loadings in Table A2, represented all three variables to a comparable degree, and captured a proportion of 0.82 of their variance.

**Table A2:** Results of the Principal Component Analysis over the data of three variables relating to general musical experience.

Principal Component Analysis	Comp.1	Comp.2	Comp.3
Importance of Components			
Standard deviation	1.57	0.52	0.51
Proportion of Variance	0.82	0.09	0.09
Loadings			
Duration of musical training	0.58	0.81	0.12
Number of musical instruments/activities	0.58	−0.50	0.64
Age of musical instrument/activity acquisition	0.58	−0.30	−0.76

## Appendix C

For each group, we calculated a generalized linear mixed effects model (under use of the bobyqa optimizer) with condition as fixed factor, participants and items as random factors, but no random slopes because of false convergence. The intercept was set to zero, so that each of the three acoustic conditions (intensity, control, and duration) was compared to chance. Results are provided in Table A3.

**Table A3:** Results of two generalized mixed effects models (one for the group of adults with dyslexia, and one for the group of adults without dyslexia) to test the groups’ preferences against chance in the three acoustic conditions reported in Section 3.3.

Fixed Effects	*β*	*SE*	*z*	*p*	*Sig.*
**With Dyslexia**					
Intensity	0.41	0.07	5.71	<0.001	***
Control	0.31	0.14	2.14	0.03	*
Duration	−0.25	0.07	−3.58	<0.001	***
**Without Dyslexia**					
Intensity	1.12	0.12	9.24	<0.001	***
Control	0.92	0.18	5.16	<0.001	***
Duration	−0.53	0.11	−4.68	<0.001 ^1^	***

^1^ Formula: Response ~ −1 + Condition + (1 | participant) + (1 | item). Each line shows the coefficients of the intercept of each of the separate models. Negative β estimates indicate more iambic responses, and positive β estimates indicate more trochaic responses. Level of significance: * *p* < 0.05, *** *p* < 0.001.

## Appendix D

The results of the linear regression analysis for testing whether musical rhythm perception ability (MET scores) is predicted by dyslexia (group factor), cognitive ability (first principal component of the WAIS scores) and musical experience (first principal component combining the number of learned musical instruments/activities, age of musical acquisition, and duration of musical training) are provided in Table A4.

**Table A4:** Parameters of the regression analysis of the effects of dyslexia, musical experience and cognitive ability on musical rhythm perception ability.

*Effects*	*β*	*SE*	*T*	*p*	*Sign.*
Intercept	−0.01	0.02	−0.65	0.52	
Group	0.07	0.03	2.28	0.03	*
Musical experience	−0.01	0.01	−1.10	0.28	
Cognitive ability	0.03	0.01	3.27	0.002	**
Group* Musical rhythm perception ability	0.02	0.02	1.44	0.16	
Group*Cognitive ability	0.01	0.02	0.63	0.53 ^1^	

^1^ Formula: lm(Musical rhythm perception ability ~ Group*Musical experience + Group*Cognitive ability). Level of significance: * *p* < 0.05, ** *p* < 0.01.

## Appendix E

For each group, we calculated a generalized linear mixed effects model (under use of the bobyqa optimizer) with condition as fixed factor, participants and items as random factors, but no random slopes because of false convergence. The intercept was set to zero, so that each of the three acoustic conditions (intensity, control, and duration) was compared to chance. Results are provided in Table A10. To test the effects of the factors group and musical rhythm perception ability on the three conditions, while controlling for cognitive ability and musical experience, generalized linear mixed-effects models were built that incrementally increased the number of predictors in a stepwise fashion. Model fits were compared by means of their loglikelihood using the anova() function from the LME4 package ([57]). Successive difference contrast coding was used for comparing groups and conditions. This contrast (coded with the contr. sdif() function from the MASS package, [85]) assigns the grand mean to the intercept, and beta coefficients indicate the difference scores between two compared levels. In the case of group, this contrast was specified as subtracting the group with dyslexia from the group without dyslexia. For condition, the contrasts were Duration-Intensity (β reflecting duration minus intensity) and Control-Duration (control minus duration). Both continuous predictors were centered around their mean to reduce collinearity, and z-transformed (using the scale() function) as models with untransformed predictors did not converge (mathematically, z-transformation does not affect the results). The models were coded containing a fraction, with condition being the numerator, and the predictors group, musical rhythm perception ability, and/or cognitive ability being the denominator. By this, it is possible to assess the effects of the predictors group, musical rhythm perception ability and cognitive ability on each of the conditions in separation.

Random intercept for participants and items, and random slopes for the condition contrasts by participants were included. Correlations of the random slopes by participants were subtracted (by ||), as not all reported models would converge when including them and comparisons suggested that they did not significantly account for variance. Models including random slopes for cognitive ability, group, and/or musical rhythm perception ability by item did not improve the model fits.

Parameters of the tested models are reported in the tables below. To correct for multiple comparisons, adjusted *p*-values (Bonferroni correction) are reported in the last column (model 2, *p*-values multiplied by 2, model 3: *p*-values multiplied by 3, etc.). Level of significance: * *p* < 0.05, ** *p* < 0.01, *** *p* < 0.001.

**Table A5:** Output of the first model exploring the effects of group on the three conditions.

**Random Effects**	**Groups**	**Name**		**Variance**	**Std.Dev.**
	Item	(Intercept)		0.15	0.38
	Participant	Control-Duration		1.07	1.03
	participant.1	Duration-Intensity		0.09	0.30
	participant.2	(Intercept)		0.00	0.00
**Fixed Effects**	***β***	***SE***	***z***	***p***	***Sign.***
(Intercept)	0.36	0.06	5.65	<0.001	***
Duration-Intensity	−1.26	0.17	−7.27	<0.001	***
Control-Duration	1.04	0.13	8.29	<0.001	***
Intensity*Group	0.75	0.25	3.00	0.003	**
Duration*Group	−0.28	0.17	−1.66	0.10	
Control*Group	0.60	0.25	2.42	0.02	* ^1^

^1^ Formula: *Response* ~ *Condition*/(*Group*) + (1 + *condL2v1* + *condL3v2* || *participant*) + (1 | *item*). Level of significance: * *p* < 0.05, ** *p* < 0.01, *** *p* < 0.001.

**Table A6:** Output of the second model exploring the effects of musical rhythm perception ability on the three conditions.

**Random Effects**	**Groups**	**Name**		**Variance**		**Std.Dev.**
	Item	(Intercept)		0.14		0.37
	Participant	Control-Duration		1.10		1.05
	participant.1	Duration-Intensity		0.10		0.32
	participant.2	(Intercept)		0.00		0.01
**Fixed Effects**	***β***	***SE***	***z***	***p***	***Sign*.**	**Corrected *p***
(Intercept)	0.37	0.07	5.54	<0.001	***	<0.001
Duration-Intensity	−1.26	0.17	−7.21	<0.001	***	<0.001
Control-Duration	1.06	0.13	8.37	<0.001	***	<0.001
Intensity*Musical rhythm perception ability	0.29	0.13	2.34	0.02	*	0.04
Duration*Musical rhythm perception ability	−0.32	0.09	−3.72	<0.001	***	<0.001
Control*Musical rhythm perception ability	0.55	0.13	4.27	<0.001	***	<0.001 ^1^

^1^ Formula: *Response* ~ *Condition*/(*Musical rhythm perception ability*) + (1 + *Duration-Intensity* + *Control-Duration* || *participant*) + (1 | *item*). Level of significance: * *p* < 0.05, *** *p* < 0.001.

**Table A7:** Output of the third model exploring the effects of group and musical rhythm perception ability on the three conditions.

**Random Effects**	**Groups**	**Name**			**Variance**	**Std.Dev.**
	item	(Intercept)			0.14	0.38
	participant	Control-Duration			1.02	1.01
	participant.1	Duration-Intensity			0.01	0.11
	participant.2	(Intercept)			0.07	0.26
**Fixed Effects**	***β***	***SE***	***z***	***p***	***Sign.***	***Corr. p***
(Intercept)	0.40	0.07	5.53	<0.001	***	<0.001
Duration-Intensity	−1.20	0.20	−6.08	<0.001	***	<0.001
Control-Duration	0.91	0.14	6.41	<0.001	***	<0.001
Intensity*Group	0.60	0.28	2.12	0.03	*	0.09
Duration*Group	0.05	0.19	0.27	0.78		
Control*Group	0.07	0.28	0.24	0.81		
Intensity*Musical rhythm perception ability	0.14	0.14	0.97	0.33		
Duration* Musical rhythm perception ability	−0.34	0.10	−3.53	<0.001	***	<0.001
Control* Musical rhythm perception ability	0.55	0.15	3.74	<0.001	***	<0.001
Intensity*Group* Musical rhythm perception ability	−0.14	0.28	−0.52	0.61		
Duration*Group* Musical rhythm perception ability	−0.40	0.19	−2.09	0.04	*	0.12
Control*Group* Musical rhythm perception ability	0.20	0.29	0.67	0.50 ^1^		

^1^ Formula: *Response* ~ *Condition*/(*Group * Musical rhythm perception ability*) + (1 + *Duration-Intensity* + *Control-Duration* || *participant*) + (1| *item*).Level of significance: * *p* < 0.05, *** *p* < 0.001.

**Table A8:** Output of the fourth model that extends model 3 by cognitive ability as control variable, which did not improve the model fit.

**Random Effects**	**Groups**	**Name**			**Variance**	**Std.Dev.**
	Item	(Intercept)			0.15	0.38
	Participant	Control-Duration			0.91	0.96
	participant.1	Duration-Intensity			0.07	0.26
	participant.2	(Intercept)			0.01	0.08
**Fixed Effects**	***β***	***SE***	***z***	***p***	***Sign.***	***Corr. p***
(Intercept)	0.36	0.07	4.93	<0.001	***	<0.001
Duration-Intensity	−1.13	0.2	−5.77	<0.001	***	<0.001
Control-Duration	0.89	0.15	5.98	<0.001	***	<0.001
Intensity*Group	0.44	0.28	1.58	0.12		
Duration*Group	0.12	0.19	0.61	0.54		
Control*Group	0.02	0.29	0.07	0.94		
Intensity* Musical rhythm perception ability	0	0.15	0.01	0.99		
Duration* Musical rhythm perception ability	−0.29	0.1	−2.80	0.005	**	0.02
Control* Musical rhythm perception ability	0.49	0.16	2.97	0.003	**	0.01
Intensity*Cognitive ability	0.31	0.15	2.03	0.04	*	0.16
Duration*Cognitive ability	−0.12	0.11	−1.12	0.26		
Control*Cognitive ability	0.11	0.17	0.64	0.52		
Intensity*Group* Musical rhythm perception ability	−0.37	0.3	−1.22	0.22		
Duration*Group* Musical rhythm perception ability	−0.41	0.2	−2.01	0.04	*	0.16
Control*Group* Musical rhythm perception ability	0.07	0.33	0.21	0.84		
Intensity*Group*Cognitive ability	0.52	0.31	1.68	0.09		
Duration*Group*Cognitive ability	0.04	0.21	0.18	0.86		
Control*Group*Cognitive ability	0.24	0.34	0.73	0.47 ^1^		

^1^Table A8 reports the results of Model 4, which extended Model 3 by adding cognitive ability as control variable (Formula: *Response* ~ *Condition*/(*Group***Musical rhythm perception ability* + *Cognitive ability*) + (1 + *Duration-Intensity* + *Control-Duration* || *participant*) + (1 | *item*). Level of significance: * *p* < 0.05, ** *p* < 0.01, *** *p* < 0.001. Not different from Model 3, the results of Model 4 suggest highly significant effects of musical rhythm perception ability on the duration (*p* = 0.02) and the control condition (*p* = 0.008). Intensity was, just as in Model 3, not modulated by musical rhythm perception ability, and there were, again, no interactions of any condition and group, and neither any three-way interactions of any condition with group and musical rhythm perception ability. Altogether, there were no significant effects of cognitive ability. Model comparisons revealed that Model 4 was not better than Model 3 (χ^2^ = 8.05, *p* < 0.23).

**Table A9:** Output of the fifth model that extends model 3 by musical experience as control variable, which did not improve the model fit.

**Random Effects**	**Groups**	**Name**			**Variance**	**Std.Dev.**
	item	(Intercept)			0.14	0.38
	participant	Control-Duration			0.98	0.99
	participant.1	Duration-Intensity			0.06	0.25
	participant.2	(Intercept)			0.01	0.05
**Fixed Effects**	***β***	***SE***	***z***	***p***	***Sign.***	***Corr. p***
(Intercept)	0.38	0.07	5.39	<0.001	***	<0.001
Duration-Intensity	−1.14	0.2	−5.71	<0.001	***	<0.001
Control-Duration	0.85	0.15	5.76	<0.001	***	<0.001
Intensity*Group	0.62	0.28	2.19	0.02	*	0.1
Duration*Group	−0.06	0.19	−0.32	0.74		
Control*Group	0.04	0.29	0.17	0.87		
Intensity*Musical rhythm perception ability	0.12	0.14	0.88	0.38		
Duration*Musical rhythm perception ability	−0.32	0.09	−3.44	<0.001	***	0.003
Control*Musical rhythm perception ability	0.53	0.15	3.65	<0.001	***	0.001
Intensity*Musical experience	−0.03	0.13	0.23	0.81		
Duration*Musical experience	0.2	0.09	2.38	0.02	*	0.09
Control*Musical experience	0.09	0.13	0.73	0.46		
Intensity*Group*Rhythm perception ability	−0.13	0.28	−0.46	0.64		
Duration*Group*Rhythm perception ability	−0.46	0.19	−2.49	0.01	*	0.06
Control*Group*Rhythm perception ability	0.19	0.29	0.69	0.49		
Intensity*Group*Musical experience	0.14	0.25	0.56	0.58		
Duration*Group*Musical experience	−0.07	0.17	−0.43	0.66		
Control*Group*Musical experience	0.33	0.26	1.26	0.21 ^1^		

^1^ Formula: *Response* ~ *Condition*/(*Group * Muscial rhythm perception ability* + *Musical experience*) + (1 + *Duration-Intensity* + *Control-Duration* || *participant*) + (1 | *item*). Level of significance: * *p* < 0.05, *** *p* < 0.001.Table A9 reports the results of Model 5, which extended Model 3 by adding musical experience as control variable (Formula: *Response* ~ *Condition*/(*Group*Musical rhythm perception ability* + *Musical experience*) + (1 + *Duration-Intensity* + *Control-Duration* || *participant*) + (1 | *item*). The results of Model 5 match those of Model 3, suggesting the same highly significant effects of musical rhythm perception ability on the duration (*p* = 0.003) and the control condition (*p* = 0.001). Altogether, there were no significant effects of musical experience. Model comparisons revealed that Model 5 was not better than Model 3 (χ^2^ = 8.82, *p* < 0.18).

## Appendix F

The results of the linear regression analysis for testing the effects of dyslexia (group factor), musical rhythm perception ability (MET scores), and cognitive ability (First principal component of the WAIS scores) on reading ability (SLRT nonword reading scores) are provided in Table A10.

**Table A10:** Parameters of the regression analysis of the effects of dyslexia, musical rhythm perception ability and cognitive ability on reading ability.

*Effects*	*β*	*SE*	*T*	*p*	*Sign.*
Intercept	1.84	3.58	0.52	0.61	
Group	31.48	7.15	4.40	<0.001	***
Musical rhythm perception ability	18.91	33.50	0.56	0.58	
Cognitive ability	3.75	2.55	1.47	0.15	
Musical experience	2.21	2.05	1.08	0.29	
Group*Musical rhythm perception ability	−132.09	66.99	−1.98	0.056	
Group*Cognitive ability	2.89	5.11	0.57	0.58	
Group*Musical experience	4.72	4.11	1.15	0.26 ^1^	

^1^ Formula: lm(Reading ability ~ Group*Musical rhythm perception ability + Group*Cognitive ability+Group*Musical experience). Level of significance: *** *p* < 0.001.
